# Cannabinoids and Genetic Epilepsy Models: A Review with Focus on CDKL5 Deficiency Disorder

**DOI:** 10.3390/ijms251910768

**Published:** 2024-10-07

**Authors:** Sean Massey, Anita Quigley, Simone Rochfort, John Christodoulou, Nicole J. Van Bergen

**Affiliations:** 1 Brain and Mitochondrial Research Group, Murdoch Children’s Research Institute, Royal Children’s Hospital, Melbourne, VIC 3052, Australia; sean.massey@mcri.edu.au (S.M.); john.christodoulou@mcri.edu.au (J.C.); 2 Electrical and Biomedical Engineering, School of Engineering, RMIT University, Melbourne, VIC 3000, Australia; anita.quigley@rmit.edu.au; 3 Aikenhead Centre for Medical Discovery, St. Vincent’s Hospital, Melbourne, VIC 3065, Australia; 4 Centre for Clinical Neuroscience and Neurological Research, St. Vincent’s Hospital, Melbourne, VIC 3065, Australia; 5 Department of Medicine, University of Melbourne, Melbourne, VIC 3065, Australia; 6 School of Applied Systems Biology, La Trobe University, Bundoora, VIC 3083, Australia; simone.rochfort@agriculture.vic.gov.au; 7 Agriculture Victoria Research, AgriBio Centre, AgriBio, Melbourne, VIC 3083, Australia; 8 Department of Paediatrics, University of Melbourne, Melbourne, VIC 3052, Australia

**Keywords:** CDKL5, CDD, CBD, cannabidiol, cannabinoids, refractory epilepsy

## Abstract

Pediatric genetic epilepsies, such as CDKL5 Deficiency Disorder (CDD), are severely debilitating, with early-onset seizures occurring more than ten times daily in extreme cases. Existing antiseizure drugs frequently prove ineffective, which significantly impacts child development and diminishes the quality of life for patients and caregivers. The relaxation of cannabis legislation has increased research into potential therapeutic properties of phytocannabinoids such as cannabidiol (CBD) and Δ9-tetrahydrocannabinol (THC). CBD’s antiseizure properties have shown promise, particularly in treating drug-resistant genetic epilepsies associated with Lennox–Gastaut syndrome (LGS), Dravet syndrome (DS), and Tuberous Sclerosis Complex (TSC). However, specific research on CDD remains limited. Much of the current evidence relies on anecdotal reports of artisanal products lacking accurate data on cannabinoid composition. Utilizing model systems like patient-derived iPSC neurons and brain organoids allows precise dosing and comprehensive exploration of cannabinoids’ pharmacodynamics. This review explores the potential of CBD, THC, and other trace cannabinoids in treating CDD and focusing on clinical trials and preclinical models to elucidate the cannabinoid’s potential mechanisms of action in disrupted CDD pathways and strengthen the case for further research into their potential as anti-epileptic drugs for CDD. This review offers an updated perspective on cannabinoid’s therapeutic potential for CDD.

## 1. Introduction

Phytocannabinoids, products derived from the cannabis plant, are an increasingly attractive therapeutic target for the treatment of various neurological disorders. The major constituent of cannabis extracts is Δ9-tetrahydrocannabinol (THC), which is responsible for the intoxicating, psychoactive effects for which cannabis is well known. Another major cannabinoid product is cannabidiol (CBD), which is mildly psychoactive and non-intoxicating with low rates of dependency or risk of drug abuse but shows potential for diverse therapeutic uses, including anti-inflammatory [1], analgesic [2], and provides neuroprotective effects in Parkinson and Alzheimer disease [3,4]. However, more robust human trials are needed to substantiate these effects fully. The study of these cannabinoids has led to the discovery of endocannabinoid systems present in all vertebrates. The system comprises two primary cannabinoid receptors (CB1 and CB2) and endogenous cannabinoid ligands (endocannabinoids), primarily 2-arachidonoylglcerol (2-AG) and N-arachidonoylethanolamide (AEA) [5,6].

Genetic epilepsies are one of the most promising and commonly studied disease targets of CBD [7]. Currently, there is one U.S. Food and Drug Administration (FDA)-approved prescription CBD product on the market, Epidiolex, used for treating seizures associated with the developmental and epileptic encephalopathy (DEE) disorders Dravet syndrome (DS), Lennox–Gastaut Syndrome (LGS), and as an adjunctive use for Tuberous Sclerosis Complex (TSC). This product is marketed as Epidyolex in the European Union, United Kingdom, Australia and other regions and is approved as adjunctive therapy for seizures associated with DS and LGS. Additionally, there are reports of CBD attenuating seizures in patients with other drug-resistant epileptic etiologies [8].

CDKL5 Deficiency Disorder (CDD) is a rare DEE caused by variants in the Cyclin-Dependent Like Kinase 5 (CDKL5) gene [9]. Individuals with CDD experience early-onset drug-resistant seizures, with a median age-of-onset of 6 weeks (1 week–1.5 years), along with severe neurodevelopmental impairment and profound lifelong disability [10]. Frequent seizures (up to 29 per day) often present as generalized tonic or tonic–clonic seizures. At a median age of 2 years, there is often a “honeymoon period” during which individuals may be seizure-free for approximately 6 months [11,12]. Seizures often relapse, and epileptic spasms become the most common seizure type. Clonic, atonic, and absence seizures are less common [13].

Case studies have highlighted the heterogeneous nature of seizures in CDD, with reports of myoclonic seizures, hypermotor–tonic spasms, and reflex seizures [14,15,16,17]. These seizures correlate with a diverse range of electroencephalographic findings, including ictal and interictal abnormalities [14,18], which may explain the idiosyncratic effectiveness of drugs and drug combinations among CDD. 

The severe seizures experienced in DEEs can significantly impede developmental progression and diminish the quality of life for both patients and their families beyond the underlying etiology [19,20,21,22,23]. Families and caregivers have identified seizures as the second most burdensome symptom, after global developmental delay, and as a crucial factor in determining the quality of life [24].

Approximately 30% of individuals with epilepsy develop drug-resistant epilepsy [25]. CDD without epilepsy is rare [13,26], and most CDD cases develop drug-resistant epilepsy [12,27]. Approximately 15% of individuals with CDD use a single anti-epileptic drug (AED), 25% use two AEDs, 44% use three or more AEDs, and 9% use no AEDs [19,28]. This is broadly in line with AED use in patients with drug-resistant epilepsy from a variety of other etiologies [29]. There is debate in the field about the effectiveness of introducing multiple AEDs, which rarely reduce seizure frequency but increase side effects [30,31,32]. Individuals with CDD taking three or more AEDs were perceived to have a worse quality of life [19]. Twenty percent were currently using medical cannabis, although the source or method of use is unknown [19].

Common side effects of AEDs in DEE individuals include gastrointestinal upset, somnolence, decreased appetite, fatigue, skin rashes, and fever [33,34]. More severe adverse effects include depression, respiratory tract infections, aplastic anemia, liver failure, cardiovascular risks, cognitive decline, and paradoxically induced seizures [33,34,35]. The most common AEDs for CDD-related epilepsy are clobazam (CLB), valproate, phenobarbital, topiramate, vigabatrin, and levetiracetam [12]. The mechanism of action of many of these AEDs is unknown or only partly understood. Alternative treatments include ketogenic diets, corticosteroids, and neural stimulation therapies [12]. However, this polytherapy approach can also significantly impact the quality of life, reduce adherence to treatment, and cause behavioral issues [36]. Consideration of adverse effects and the overall quality of life is pivotal for the selection of therapeutics, especially for the treatment of pediatric patients.

Side effects of CBD-based therapies are usually mild and can include somnolence, fatigue, vomiting, diarrhea, decreased appetite, and fever [37]. More serious adverse events include liver damage, pneumonia, cognitive and behavioral disorders, and increased incidence of seizures [37,38]. Most safety data on chronic CBD usage have been gathered from studies in adults, with limited information available on its long-term effects in pediatric populations. However, the potential side effects must be weighed against the debilitating epilepsy experienced by many CDD patients. Recent reviews offer comprehensive summaries of CBD’s safety and potential side effects [39,40].

The use of cannabinoids in Western medicine for convulsive disorders dates back to the mid-1800s, including in pediatric cases [41]. More recently, the societal acceptance and legalization of medicinal cannabis in the last decade has seen greater uptake and has advanced research due to ease of access, increased demand, and improvements in cultivation, extraction, and purification.

Researching the antiseizure properties of CBD in pediatric populations presents several challenges, including the major challenge of a lack of standardization of CBD and cannabinoid products. Often, CBD and THC concentrations are incorrectly reported [42,43]. Additionally, the other 120 cannabinoid compounds and other trace compound groups, such as terpenes, are overlooked in the labeling and analysis of specific cannabis extracts [44,45,46]. These compounds can appear in trace amounts in CBD extracts [47], and they could provide synergistic effects [48]. Additionally, cannabinoid concentrations in plant extracts vary based on the specific cannabis cultivar, extraction methods, plant age, and propagation techniques [49]. The lack of consistency of trace compounds complicates comparisons between and within trials, making it difficult to obtain high-quality efficacy, toxicity, and dose-response data. Even pharmaceutical-grade CBD contains detectable levels of THC, which may exceed some recommended safety limits of 0.021 mg/day and is unlikely to remain consistent from one preparation to the next [43].

Currently, there are no double-blinded placebo-controlled clinical trials specifically exploring the use of CBD for treating CDD-associated epilepsy. Clinical trials in CDD have multiple challenges, including a relatively small cohort of affected individuals and variability in clinical phenotype and disease progression. The seizure-free honeymoon period and development of tolerances require long-term follow-up and consistent record-keeping, which can be a burden on participants’ caregivers. In addition, with the rising popularity of CBD, individuals who may have already tried CBD, either off-label or self-medicated, may be excluded from participation in future trials, further limiting the pool of individuals eligible for clinical trials. 

There is already a wide spectrum of therapeutics used in CDD patients with treatment-resistant epilepsy, and these concomitant AEDs and their combined synergistic effects should be included in the analysis. Some AEDs have the potential to increase adverse effects when paired with CBD [34,50,51] and can change the metabolism of the drugs [50,51]. CBD, CLB, and other drugs share a catabolism mechanism through the cytochrome (CYP) pathway. In pediatric patients, CBD increases CLB catabolism to nCLB by inhibiting the CYP 2C19 and CYP 3A4 enzymes. While nCLB is more potent than CLB itself, reducing CLB dosage minimizes adverse effects while maintaining seizure control. Interestingly, CLB does not appear to affect CBD metabolism [50]. Elevated serum levels of nCLB, topiramate, and rufinamide in pediatric patients have been observed after CBD administration [51]. While topiramate is metabolized by the CYP family, rufinamide is not, making the effect of CBD on rufinamide levels unclear. The authors hypothesize that interactions with the sesame oil vehicle in CBD products could add further complexity to CBD’s interactions with other anti-epileptic drugs (AEDs). Additionally, co-administration of CBD and valproate was associated with elevated aspartate aminotransferase (AST) and alanine aminotransferase (ALT) levels, suggesting liver toxicity. Four of 14 of these individuals regained normal liver function when using CBD alone.

In lieu of blinded placebo-controlled studies, researchers have opted for open-label studies or observational studies, although both have challenges. Regardless, studies show promising results in trialing CBD for CDD, suggesting the need for further high-quality trials to determine efficacy.

Model organisms and preclinical models can be valuable tools for studying the anti-epileptic effects of cannabinoids and understanding general mechanisms of action, particularly for drug-resistant epilepsies. These models allow the exploration of different CBD and cannabinoid concentrations, formulations, processing methods, and interactions with other AEDs, which can help establish guidelines on dosing or toxicity and elucidate pharmacodynamics, pharmacokinetics, and drug-drug interactions. There is potential for idiosyncratic polytherapy combinations to be developed through patient- or genetic variant-specific models; however, with over 30 approved AEDs, specific combinations for each patient are challenging to elucidate. Preclinical model systems are ideal for focusing the outcomes of future trials but require robust disease-specific models accurately mimicking human disease, posing a dilemma around the most effective model systems to utilize. Below, we provide an overview of the endocannabinoid system, highlight overlapping pathways with CDKL5, provide an overview of CDD models, and speculate on the utility of CBD treatment in CDD and other genetic epilepsies. 

## 2. The Endocannabinoid System and Its Role in Epilepsy and CDKL5 Deficiency Disorder

**Figure 1:**
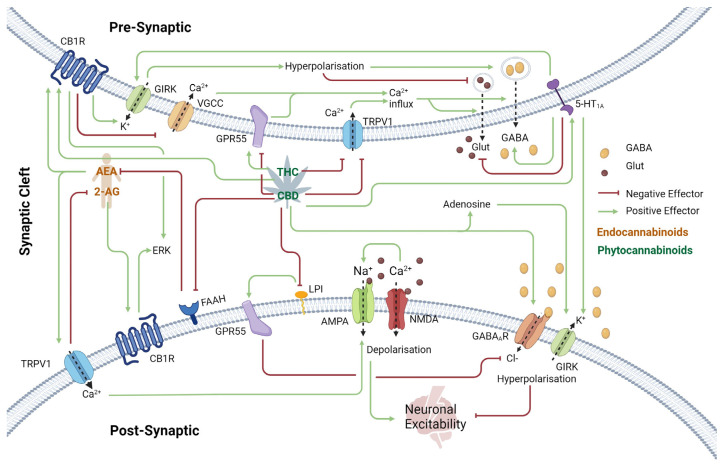
Schematic of the Endocannabinoid System at the Synapses in the Cerebral Cortex. The endogenous Endocannabinoids AEA and 2-AG act as retrograde neurotransmitters that activate CB1R. Presynaptic CB1R activation leads to GIRK channel opening, promoting K^+^ efflux, hyperpolarizing the presynaptic membrane, and reducing neuronal excitability by simultaneously preventing the release of the excitatory neurotransmitter glutamate and promoting the release of the inhibitory neurotransmitter GABA. CB1R activation also inhibits some VGCCs, reducing Ca^2+^ influx. Since Ca^2+^ is required for neurotransmitter release into the synaptic cleft, this results in an overall inhibitory effect on synaptic transmission. 5-HT1A receptors also activate GIRKs and inhibit VGCC response to stimulation by CBD, also reducing neuronal excitability. Additionally, 5-HT1A modulates presynaptic vesicle release by inhibiting the adenylyl cyclase pathway, further dampening neuronal transmission. TRPV1, a non-selective cation channel, is activated by the endocannabinoids AEA, CBD, and THC. However, prolonged exposure to plant-derived phytocannabinoids desensitizes TRPV1, preventing activation by endogenous ligands. In both the pre- and postsynaptic regions, TRPV1 enhances neuronal excitability through Ca^2+^ influx and depolarization of the neuronal membrane. Therefore, CBD has an overall inhibitory effect on synaptic transmission. AMPA-R are ion channels responsible for the majority of fast excitatory neurotransmission in the brain. The binding of glutamate to AMPA-R on the postsynaptic membrane leads to Na^+^ influx and depolarization. CBD inhibits FAAH, the enzyme responsible for AEA breakdown, leading to AEA accumulation in the synaptic cleft and enhancing its inhibitory effects. Additionally, CBD interacts with the adenosine system by preventing adenosine reuptake, which enhances neuronal inhibition via GIRK activation and inhibition of VGCC and adenylyl cyclase pathways. GABA_A_R is the primary target of GABA. As a Cl^−^ channel, its activation by GABA results in postsynaptic membrane hyperpolarization, inhibiting neuronal excitability. Conversely, the excitatory neurotransmitter glutamate binds to NMDA-R, depolarizing the postsynaptic membrane and increasing neuronal excitability. Abbreviations: Receptors; CB1R (Cannabinoid Receptor 1), GIRK (G-protein-coupled inwardly rectifying potassium), VGCC (Voltage-Gated Calcium Channel), TRPV1 (transient receptor potential cation channel subfamily V member 1), GPR55 (G protein-coupled receptor 55), AMPA-R (α-amino-3-hydroxy-5-methyl-4-isoxazolepropionic acid receptor), NMDA-R (N-methyl-D-aspartate Receptor), GABA_A_R (γ-aminobutyric acid Receptor A), FAAH (Fatty acid amide hydrolase), 5-HT1A (5-hydroxytryptamine 1A Receptor), Endocannabinoids; AEA (Anandamide), 2-AG (2-Arachidonoylglycerol), Phytocannabinoids; THC (Δ9-tetrahydrocannabinol), CBD (Cannabidiol), Neurotransmitters; GABA (γ-aminobutyric acid), Glut (Glutamate). Created in BioRender [54].

A key aspect of effective therapeutic development is understanding the underlying cause of a particular disorder, as well as the mechanism of action of the drug of interest. Here, we summarize CBD’s effects on the brain and examine its overlap with current research into CDKL5 function. There are many excellent recent reviews that provide an in-depth general discussion on CBD and endocannabinoid function [52,53]. The endocannabinoid system (ECS) is a complex cell-signaling system that plays a vital role in maintaining homeostasis in the body (Figure 1). The ECS is composed primarily of two neuronal inhibitory G-protein-coupled receptors (GPCRs): the cannabinoid type 1 receptor (CB1R) and the cannabinoid type 2 receptor (CB2R), and two main endocannabinoid retrograde agonists, anandamide (AEA) and 2-arachidonoylglycerol (2-AG). Much of the ECS is an ongoing area of research, and the function of each component can depend on the brain region or cell type.

### 2.1. Cannabinoid Receptor 1 (CB1R) in the Central Nervous System

CB1 receptors (CB1R) are predominantly found in the central nervous system (CNS), especially in regions such as the hippocampus, basal ganglia, and cerebellum [55], where they play a critical role in modulating neurotransmitter release. CB1R activates ion channels such as G-protein-coupled inwardly rectifying potassium channels (GIRKs) through the Gi/o protein pathway [56]. GIRKs hyperpolarize the presynaptic membrane, inhibiting the release of the excitatory neurotransmitter glutamate and promoting the release of the inhibitory neurotransmitter GABA into the synaptic cleft (Figure 1). CB1R also inhibits N- and P/Q-type voltage-gated Ca^2+^ channels (VGCC), preventing an influx of Ca^2+^ into presynaptic regions. Ca^2+^ is required for the release of neurotransmitter presynaptic vesicles. 

THC acts as an agonist of CB1R [57], while CBD has a low binding affinity to CB1R [58]. 

### 2.2. Transient Receptor Potential Vanilloid 1 (TRPV1) Receptor

Transient Receptor Potential Vanilloid 1 receptors (TRPV1) are non-selective cation channels involved in the regulation of pain perception and neuronal excitability. They are activated by various stimuli, including heat, protons, and ligands such as AEA, THC, and CBD [59,60]. Upon activation, TRPV1 allows the influx of Ca^2+^ and Na^+^ ions, which can lead to increased neuronal excitability through glutamate release [61,62]. However, prolonged activation by THC or CBD can desensitize TRPV1, reducing its responsiveness to endogenous ligands and potentially diminishing pain signaling [63,64,65].

**Figure 2:**
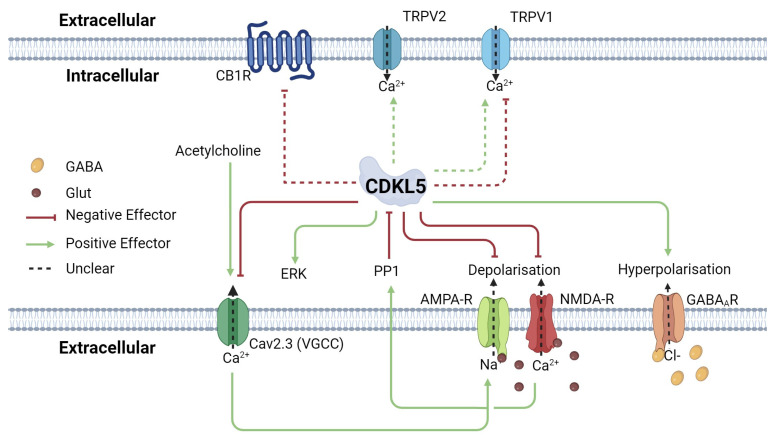
Schematic of CDKL5 interactions on cannabinoid targets. NMDA-R mediates the translocation of CDKL5 to the cytoplasm via PP1, which promotes CDKL5 degradation by accumulation in the cytoplasm. CDKL5 also regulates the localization of NMDA-R and AMPA-R by promoting their removal from the postsynaptic membrane. Cav2.3 is a voltage-gated calcium channel important for depolarization of the postsynaptic membrane. The resultant Ca^2+^ also positively affects NMDA-R. CDKL5 downregulates Cav2.3 by direct phosphorylation. CB1R, TRPV1, and TRPV2 levels are affected in *cdkl5*^−/y^ KO mice; however, the mechanism is tissue-specific and still unknown. Abbreviations: Receptors: CB1R (Cannabinoid Receptor 1), VGCC (Voltage-Gated Calcium Channel), TRPV1 (transient receptor potential cation channel subfamily V member 1), TRPV1 (transient receptor potential cation channel subfamily V member 2), GPR55 (G protein-coupled receptor 55), AMPA-R (α-amino-3-hydroxy-5-methyl-4-isoxazolepropionic acid receptor), NMDA-R (N-methyl-D-aspartate Receptor), GABA_A_R (γ-aminobutyric acid Receptor A) Neurotransmitters: GABA (γ-aminobutyric acid), Glut (Glutamate). Created in BioRender [68].

In the context of CDD, TRPV1-mediated signaling is disrupted (Figure 2). CDKL5 interacts with TRPV1 in pain pathways, and disruptions in dorsal root ganglia have been observed in animal and induced pluripotent stem cell (iPSC) models [66]. Many individuals with CDD exhibit reduced pain perception [66]. Furthermore, studies have shown significant alterations in the expression levels of hippocampal CB1R and TPRV1 and cortical TRRV1 and TRPV2 expression in adult CDKL5 R59X mutant mice, suggesting dysregulation of endocannabinoid signaling, which may coincide with the hyperexcitability phenotype in CDD [67]. This gives a promising mechanism of action for CBD to reduce seizures in CDD.

### 2.3. Excitatory and Inhibitory Neurotransmission Receptors

NMDA receptors (NMDA-R) are a primary receptor of the excitatory neurotransmitter, glutamate, which plays a critical role in excitatory synaptic transmission. When activated by glutamate in the synaptic cleft, NMDA-R allows the influx of Ca^2+^, Na^+^, and K^+^ ions, contributing to excitatory postsynaptic potentials. Excessive NMDA-R activation can lead to hyperexcitability and seizures. Cannabinoids, particularly through CB1R, indirectly reduce NMDA-R-mediated excitatory transmission by inhibiting glutamate release at presynaptic terminals. 

Glutamate stimulation of NMDA-R enhances the translocation of CDKL5 from the nucleus to the cytoplasm via protein phosphatase 1 (PP1)-mediated dephosphorylation and promotes CDKL5 degradation (Figure 2) [69,70]. Conversely, NMDA-R subcellular location is also regulated by CDKL5 in the hippocampus. In the *cdkl5*^−/y^ KO mouse model, NMDA-R accumulated at the postsynaptic terminal [71]. Treatment with a specific NMDA-R blocker attenuated hyperexcitability in these mice. However, NMDA-R dysregulation was not seen in the *cdkl5*^−/y^ rat model [72]. This disparate response in a similar model system highlights the importance of selecting models for investigation and careful consideration of how results are applied across systems.

AMPA receptors (AMPA-R) are also glutamate-responsive ion channels, primarily responsible for Na^+^ influx across the postsynaptic membrane, leading to depolarization. AMPA-R is also influenced by the influx of Ca^2+^ via NMDA-R and TPRV1, promoting its localization to the postsynaptic membrane (Figure 1) [73].

Hippocampal AMPA-R is elevated in the *cdkl5^R59X^* mouse model [74]. Acute treatment with a specific AMPA-R blocker rescued social deficits, working memory impairments, and seizure susceptibility. Likewise, AMPA-R dysregulation was not observed in *cdkl5*^−/y^ KO rats [72]. Rodent CDD models do not present with the typical early-onset epilepsy of CDD patients, but seizures appear later into adulthood [75]. Therefore, the dysregulation of NMDA-R and AMPA-R may appear later in the longer-lived rat model, but further investigation is required.

CBD also acts as a positive allosteric modulator of the inhibitory GABA_A_ receptor (GABA_A_R) [76]. GABA_A_R is a Cl^−^ channel that mediates the inhibitory effects of the neurotransmitter GABA by Cl^−^ influx at the postsynaptic terminal (Figure 1), resulting in hyperpolarization and inhibition of neuronal hyperexcitability. CDKL5 is thought to regulate GABA_A_R localization through interactions with the scaffold proteins gephyrin and collybistin. CDKL5 is already known to interact with and phosphorylate other scaffold proteins for AMPA-R regulation [77]

### 2.4. Other G-Protein-Coupled Receptors

G protein-coupled receptor 55 (GPR55) has been shown to increase intracellular Ca^2+^ levels from intracellular stores in response to endogenous agonist lysophosphatidylinositol (LPI) [78,79], depolarizing the presynaptic membrane and promoting the release of glutamate (Figure 1). GPR55 activation also downregulates postsynaptic GABA_A_R through endocytosis [80]. CBD acts as an antagonist of GPR55 by inhibiting LPI binding and thus reducing both pro-excitability pathways [80,81]. This inhibition is thought to contribute to CBD’s anticonvulsant effects. CDKL5 is also involved in GABA_A_R localization to the postsynaptic membrane [82] through its interaction with the inhibitory scaffolding complex [82].

Interestingly, the dual control of GABA-mediated inhibition by GPR55 may explain some instances of drug-resistant epilepsy. Benzodiazepines, such as clobazam, are a first-line AED used in the treatment of pediatric epilepsy. They act as an allosteric modulator of the GABA_A_R, increasing its sensitivity to GABA and enhancing inhibition [83]. However, if GPR55 modulates GABA_A_R by removing it from the postsynaptic membrane via control of endocytosis, benzodiazepines would be ineffective. By inhibiting GPR55 activation, CBD may re-establish GABA_A_R to the postsynaptic membrane, reopening the pathway to benzodiazepines. Both observational trials and the DS mouse model support this notion, where CBD in combination with benzodiazepines attenuated seizures more than benzodiazepines alone [84,85,86]. It would be interesting to see data regarding benzodiazepines-CBD efficacy in CDD patients and if CBD can rectify CDKL5-mediated GABA_A_R localization disruption. 

THC and AEA are partial agonists of GPR55, enhancing excitatory neurotransmission, contrary to the function seen with other members of the endocannabinoid system. This contradiction may be why THC can have proconvulsant effects in certain circumstances and highlights the importance of correct dosing and concentrations of medical cannabinoid extracts.

Additionally, CBD also interacts with the serotonin receptor 5-HT1A, a GPCR involved in the regulation of mood, anxiety, and neuronal excitability (Figure 1). Activation of 5-HT1A receptors by CBD leads to the hyperpolarization of neurons, reducing their excitability through the activation of GIRKs. 

### 2.5. Neurotransmitter Modulation: Adenosine and Anandamide

Finally, CBD also exerts significant effects on the adenosine system (Figure 1), which is crucial for its anticonvulsant properties. Adenosine is an inhibitory neurotransmitter that modulates synaptic activity by activating A1 receptors (A1R), leading to decreased neuronal excitability. CBD enhances adenosine signaling by inhibiting its reuptake [87,88], therefore prolonging its presence in the synaptic cleft and increasing its inhibitory effects. This mechanism is particularly relevant in the context of epilepsy, where increased adenosine signaling may help prevent seizure onset.

In addition, CBD inhibits the enzyme fatty acid amide hydrolase (FAAH) (Figure 1), which is responsible for degrading AEA. By inhibiting FAAH, CBD increases anandamide levels, enhancing its effects on CB1R and other targets and inhibiting synaptic transmission.

The endocannabinoid system is a multifaceted and highly regulated network that plays a crucial role in maintaining synaptic balance and preventing hyperexcitability, which is key to managing epilepsy and conditions like CDD. While much remains to be understood, the interaction between cannabinoids like CBD and various components of the broader endocannabinoid system offers promising avenues for therapeutic intervention. Importantly, CDKL5 interacts with and regulates key proteins of this system (Figure 2), which is hypothesized to cause the severe seizure phenotype of CDD. The growing body of evidence supporting cannabinoids as a potential AED in CDD is highly promising, as we summarize below, and should encourage continued research into further understanding mechanisms and more in-depth clinical trials. Understanding the potential dysregulation in CDD will be essential for developing effective and targeted treatment.

## 3. Human Cannabidiol Trials

Despite the promising nature of CBD in other genetic epilepsies, few clinical trials are focusing on CBD in CDD. Most reports come from anecdotal, retrospective studies. Several open-labeled CBD trials included CDD, among other etiologies, but did not conduct a CDD-specific analysis [34,86,89,90].

One observational study of a large cohort of CDD patients showed that 61 out of 168 (36%) individuals had been treated with cannabis-derived products [91]. When the product was specified, either Epidiolex (CBD) (*n* = 19) or non-approved/unspecified cannabis-derived artisanal products were used (*n* = 18). For the remaining cases, the cannabis product was unspecified. Epidiolex had a known response in 14 patients. At a two-week follow-up, four patients had a reduction in at least one seizure type by 50%, reducing to three patients over 3 months. In comparison, artisanal products showed that two out of four had reduced seizures across both time periods. Epidiolex resulted in worsened seizures in four patients, and six patients showed no response. Compared to artisanal products, one patient had increased seizure activity, and two had no response. While these are self-assessments with a small sample size, it is interesting that Epidiolex appeared to perform worse than artisanal products, perhaps due to the entourage effect, which proposes that various compounds found in cannabis-derived extracts work synergistically to create distinctive and effective benefits. There is potential that the interplay between multiple cannabinoids, including between CBD and THC, can be proconvulsant, highlighting the importance of accurate concentration testing [92]. There could also be bias due to a placebo effect where there is a larger distrust of pharmacotherapy-based compounds compared to what is seen as “natural” or “artisanal” cannabis products. Being an uncontrolled study, it is worth noting that these perceived effects align with high placebo effect rates reported in other CBD studies [93,94]. As an observational study, accurately tracking seizure frequency is challenging without daily journals, which is typically required by rigorous clinical trials. It is also difficult to obtain data on concentrations of active ingredients of CBD or more detailed and controlled information regarding patient dosing/ treatment. Additionally, there are other clinical endpoints, such as perceived pain, that may be worth assessing in future studies in CDD patients, as CBD drugs may have effects on other clinical features aside from seizures. 

Another international observational study involving 312 CDD individuals [95] reported that 82 (26%) had tried cannabis derivatives at some point, mostly CBD (88%), THC (5.4%), or both (6.8%). Of those patients who had been treated with CBD only, 55% accessed it through a prescription from a healthcare professional. Among the 70 families with available data, the caregivers of 54% (*n* = 38) of CDD patients perceived cannabinoids to improve seizure control, 24% (*n* = 17) perceived no effect on seizures, and 4.3% (*n* = 3) perceived worsening seizure frequency. These results broadly align with observations from other studies of CBD use in CDD and other genetic epilepsies [96]. Regarding adverse effects, only 12% reported what would be considered typical adverse effects, which is below the typical rate of CBD treatment [96]. A group of 30 respondents who initially did not report cannabinoid use for their children were actively using a cannabinoid product in the follow-up questionnaire. The median duration between questionnaires was 49 months. There was no difference in the median number of seizures or the number of anti-epileptic drugs (AEDs) used in this period. At a 24-month follow-up of 10 families who were using cannabinoids at the time of the initial questionnaire and perceived an improvement in seizure frequency, 64% (*n* = 7) remained on cannabinoids and continued to report lasting seizure reduction. The remaining three families had ceased cannabinoid treatment. Although these are perceived or self-reported effects, the antiseizure benefits of CBD broadly align with other genetic epilepsy research [96]. These observational studies are limited by participation bias and self-reported assessments. However, there was little difference in seizure frequency or the number of AEDs taken between active users of cannabinoids and those not currently using.

Similarly, in a prospective cohort study of a standardized CBD-enriched cannabis extract as a pediatric adjuvant treatment for DEE, one CDD patient was reported who experienced a 50–74% reduction in seizure frequency after 20 months [97]. This was comparable to the reduction in seizures observed in patients with DS. Of the 59 patients with various clinical etiologies, 78% of patients had a 50% reduction in seizure frequency, and 12% were seizure-free after CBD treatment. The observed adverse effects were considered mild or moderate, with 29% discontinuing treatment due to lack of response, increased seizure frequency, or other adverse effects.

Only one open-labeled study of CBD therapy included an analysis specifically for CDD [98]. Epidiolex was used as an adjuvant therapy for seizure disorders in 55 patients across four epileptic encephalopathies, including 18 CDD patients. The most common adverse events were diarrhea (29%), acne (22%), fatigue (22%), decreased appetite (20%), convulsion (18%), vomiting (18%), respiratory tract infection (16%), weight loss (9%), status epilepticus (5%), irritability (7%), and pyrexia (7%). Four (7%) of the 55 participants withdrew due to adverse events. CBD was shown to reduce convulsive seizure frequency in all etiologies from baseline by week 12 by 51.4%, with slight improvements extending up to week 48. Specifically, among the remaining CDD participants, the average monthly baseline seizure rate was 66%, which was reduced by 40% at week 12 (*n* = 12) and by 58% at week 48 (*n* = 11), the greatest reduction among the four DEE studied. The mean concentration of CBD for CDD patients at the final follow-up was 26.2 mg/kg/day. Concomitant AEDs were also not controlled during this trial.

### Limitations of Cannabinoid Trials

There is concern that seizure frequency is self-reported from surveys of parents or caregivers, which may introduce biases in reported outcomes. They are mostly self-selected and thus may have a positive selection bias and survivorship bias. It is also worth noting that these trials count only a proportion of seizure types, commonly convulsive seizures, as they are a distinct, reliable measure for caregivers to record, while caregivers rarely report the frequency of other seizure types, such as drop or absent seizures. There are also issues in concomitant AEDs, where the number and class used in conjunction with CBD may alter its effectiveness. Additionally, the lack of use of standardized CBD products raise issues for some studies and complicates analysis.

There is little long-term follow-up data on CBD use in CDD, with the longest observation period being 49 months [95]. There is a significant period with the use of AEDs for CDD whereby, at first, the AED appears effective, but tolerance is built up, and seizure frequency increases over time. CBD’s efficacy may be over-reported due to predominantly short-term studies being undertaken. In one study of 26 epileptic patients, including CDD (*n* = 5), only 26.9% of all patients were continuing CBD use after 2 years [99]. Three of the CDD patients had withdrawn due to lack of efficacy between 8 and 23 months, and one patient withdrew at 21 months due to severe weight loss. The remaining individual continued with CBD and experienced a >50% reduction in seizures after 4 years. Six individuals with other etiologies sustained a reduction of >50% in seizures over the four years [99].

Given the clear promise of CBD use in genetic epilepsies and its promise for seizure reduction in CDD, further research is required. The use of relevant and accurate disease models of CDD will allow accurate and reproducible testing of CBD and other related compounds in detail. Below we summarize the relevant CDD model systems, the limited knowledge of CBD studies specifically in CDD models, and extend our discussion to encompass genetic epilepsy model systems where CBD has been trialed, as this has relevance to CDD.

## 4. Epilepsy Model Systems

Preclinical model systems are essential to effectively guide the resources invested in clinical trials. They can help with drug discovery, optimize dosing, inform biomarkers to observe, and adverse effects to monitor, ensuring a more efficient, target, and ethical approach to clinical research. The exact mechanisms by which CDKL5 causes severe early-onset epilepsy are poorly understood. Altered synaptic function, dysregulated ion channels, and impaired signaling pathways contribute to increased neuronal excitability and seizure susceptibility [100,101,102]. CDKL5 is crucial for regulating various aspects of neuronal development and synaptic plasticity [77,103], and CDKL5 deficiency likely disrupts normal brain development and function, ultimately resulting in seizure generation. A major problem in drug discovery is the lack of preclinical models that both recapitulate the patient-specific biology and epilepsy phenotype and, as an added benefit, are amenable to high-throughput drug screening. 

### 4.1. Zebrafish as a Model of CDD and Cannabinoid Use

Zebrafish, due to their high fecundity and easy care, enable the rapid generation and characterization of multiple genetic models and the availability of many reporter lines of interest [104,105,106]. They also have similar molecular pathways in the brain, making them valuable tools for researching the genetic bases of epilepsy. Zebrafish models are particularly well-suited for in vivo studies of neuronal development and high-throughput analysis methods. Large-scale production is feasible, and automated tracking systems provide an unbiased assessment of performance, while optical clarity facilitates efficient live imaging, particularly as embryos and larval stages [105,107]. These qualities are advantageous in experiments aimed at identifying modifiers of disease phenotypes through the screening of large drug libraries. The development of the vertebrate brain and spinal cord, and many associated signaling pathways, are highly conserved across species. 

Zebrafish *cdkl5* KO models often recapitulate the CDD phenotype, displaying microcephaly, motor neuron defects, and impaired motor function [105,106]. Spontaneous seizures are also observed, further establishing their utility in preclinical AED trials [105]. Although cannabinoids have not yet been investigated in CDD zebrafish models, the endocannabinoid system in zebrafish has been studied in a commonly used Dravet syndrome *Scn1lab^(−/−)^* models of genetic epilepsy, with cannabinoids such as CBD, THC, CBN, LN, and synthetics shown to reduce seizure-like activity [108]. Both CBD and THC were shown to be effective in attenuating seizures in the genetic epilepsy *GABRA1^(−/−)^* model [109]. Observing synergistic effects between THC and CBD reduced the concentrations required to control seizure activity, thus lowering the incidence of adverse effects.

Zebrafish represent a valuable preclinical tool, with several available CDKL5 epilepsy models, potential for high-throughput screening, and a positive response to cannabinoids. Although they are vertebrates with genetic and physiological conservation, their distant relationship to humans means molecular drug screens may yield both false positives and false negatives, and there are some limitations in the translation of results to human studies.

### 4.2. Rodent as a Model of CDD and Cannabinoid Use

Rodent epilepsy models have been developed that provide valuable insights into epilepsy mechanisms and CBD treatments [110,111]. Rodents generally have more conserved pathways to humans than zebrafish, offering important information on disturbed neurobiology in epilepsy [112]. However, there are still significant differences between human and rodent brains, which do not adequately recapitulate human epilepsy or CDD [113], and there are differences in anti-epileptic effects and metabolism of CBD between mammal species [114].

### 4.3. CDD-Specific Rodent Models

Although the effectiveness of cannabinoids in CDD rodent models has not been extensively studied to date, several mouse *Cdkl5* models have been developed that could be used to evaluate cannabinoids [71,115,116,117]. Recently, a *Cdkl5* KO rat has also been developed [72]. Rodent *Cdkl5* KO models recapitulate several physical, behavioral, and molecular features of CDD [118,119,120,121] and exhibit learning and memory impairments, social deficits, and hyperexcitability [74]. These can be relatively straightforward endpoints for low-throughput screening but are not practical to apply to higher-throughput drug discovery approaches. 

While *Cdkl5* model rodents show abnormal epileptiform responses to electro-stimulus and chemical convulsants such as PTZ, they do not have the typical spontaneous early-onset seizures characteristic of CDD [115], likely due to unknown compensatory mechanisms in the rodent brain. However, spontaneous epileptic spasms have been observed in older mice (9–10 months) of two distinct CDKL5 models [75], although this provides challenges to high-throughput preclinical trials.

### 4.4. Effects of Cannabinoids on Seizure Frequency in Rodents 

A large-scale review gathered data from 28 studies examining the effects CBD, Cannabidivarin (CBDV), and THC have on seizure reduction [89]. In 35 discrete conditions across six species (mouse, rat, gerbil, cat, baboon, and chicken) found that THC exhibits varying effects on seizures depending on seizure type and induction method, dosing, and timing. THC reduced seizures in 57% of the conditions (20 out of 35). Conversely, THC shows proconvulsant effects, increasing seizures in 8% of the conditions (3 out of 35), and had mixed effects, both anticonvulsant and proconvulsant, in 3% (1 out of 35). THC did not significantly affect seizures in 31% of conditions (11 out of 35). The mixed effects of THC make it difficult to extrapolate data to human clinical trials. However, 83% (35 out of 42) conditions in mice and rats showed that CBD and CBDV exhibited antiseizure properties, while 16% of the conditions had no effect. CBDV also potentiated the anticonvulsant effects of phenobarbital, valproate, and ethosuximide in both rats and mice [122]. Chronic oral administration of CBD was found to be well-tolerated and reduced seizure burden, as well as improved cognitive function in a rat temporal lobe epilepsy model [123].

There has only been one study to date examining CBD treatment in CDKL5 animal models. In the *cdkl5^R59X^* mouse, CBD attenuated seizure susceptibility in response to PTZ [67] and rescued working and long-term memory impairments and social deficits present in these mice, highlighting the potential therapeutic utility of cannabinoids for CDD. In a genetic epilepsy model of SCN8A, *Scn8a^R1620L/+^*, CBD reduced PTZ and electroshock-induced seizures and improved behavioral phenotype [124]. In the mouse *Scn1^+/+^* DS model, CBD was shown to be an anticonvulsant in hyperthermia/induced seizures [92,125]. Interestingly, low-dose CBD enhanced the THC anticonvulsant ability, while at very high levels, their co-administration was proconvuslant [92]. In the Wistar Audiogenic Rat strain, a general rat epilepsy model, chronic CBD administration reduced several audiogenic seizures and attenuated CB1R expression [126].

While investigating the compensatory seizure mechanisms in CDKL5 rodent models is useful for determining potential molecular pathways for targeted therapeutics, the use of these models is less ideal for drug screening of AEDs. Because rodent epilepsy models often involve deletion variants, creating rodent models with specific human gene variant equivalents can be challenging. Overall, rodent models are less amenable to high-throughput drug screening due to time, costs, and ethical considerations involved in handling mammals.

## 5. Human iPSC-Derived Neuronal Models

### 5.1. Two-Dimensional Cell Models

The advancement of cell reprogramming and gene-editing techniques over the last decade has facilitated the investigation of disease-specific tissues, such as neurons and brain organoid models, using patient-derived cell lines specific for disease-causing mutations. Two-dimensional neuron monolayer cultures can be directly differentiated from more accessible patient samples, such as skin fibroblasts or peripheral blood mononuclear cells, or reprogrammed to induced pluripotent stem cell (iPSC) lines for directed differentiation. iPSC-derived human neurons make an excellent, disease-specific approach for first-line high-throughput drug screening to identify priority compounds with antiseizure properties and exclude ineffective compounds [127]. These can then focus research on more advanced complex models.

Despite this progress and work done modeling Dravet syndrome [128], showing that CBD increased the inhibitory potential of synaptic neurons and decreased the excitability of excitatory neurons, to our knowledge, no studies have tested the therapeutic efficacy of CBD in CDD iPSC-derived cell models. These models provide an excellent screening potential to identify more accurate data on CBD extracts and understand the pharmacodynamics of specific cannabinoid compositions and potential synergies.

### 5.2. Three-Dimensional Cell Models

Brain organoids are three-dimensional aggregates of iPSC-derived neuronal cells. They are self-assembling and have the cytoarchitecture and cell composition of the embryonic brain, overcoming many limitations of the 2D models. As with 2D systems, the 3D organoid systems can be generated from individuals with specific mutations or genetic variants can be created through genetic manipulation of the derived iPSCs. These relatively new systems provide huge benefits in human neurodevelopment and disease modeling research.

To date, several studies have used brain organoids to study CDD-associated epilepsy, showing that CDKL5 disruption leads to hyperexcitability of glutamatergic neurons and hypersynchronous neuronal activity associated with epilepsy [102,129]. Patient-derived neurospheres were utilized for high-throughput drug screening of 1112 compounds, rescuing this neural network phenotype, and this successfully identified four compounds with therapeutic potential (Ivabradine, Solifenacin, Crenigacestat, AZD1080) [102]. Endpoints examined included Ca^2+^ signaling, cell viability, and spheroid size. Additionally, other endpoints such as cilial length (Arl13b and ACIII+ immunostaining) have been identified as potential readouts for high-throughput drug screening for CDD [118] but have not yet been utilized in any high-throughput drug repurposing screens.

The human cerebral cortex is a highly complex region when compared to other animals, and cerebral organoids are one of the more highly utilized and well-characterized brain organoid models. These organoids contain functional, albeit immature, glutaminergic and GABAergic neurons [130,131]. The cerebral cortex is a brain region severely and commonly affected in genetic neurological disorders, including epilepsy, and is a brain region where CDKL5 is highly expressed [116]. 

Oxygen-glucose deprivation (OGD), along with organoid electrophysiology, can be used to model hyperexcitability. Interestingly, CBD treatment reduced OGD-induced hyperexcitability in brain organoids [132]. 

Compared to animal models, human iPSC-based cell models can offer several advantages, including more direct relevance to human biology and the reduction of ethical concerns associated with animal testing. Brain organoids give an unprecedented opportunity to explore the neurodevelopment of epilepsy, trial drug treatments, and high-throughput screening strategies. However, they also have limitations, such as the complexity of fully recapitulating in vivo conditions, lack of vascularization and blood-brain barrier, and the limits to which they recapitulate later in utero conditions and postnatal brain development. 

Looking ahead, ongoing research efforts aim to further refine and improve these iPSC-derived brain models and explore their full potential in understanding disease mechanisms and discovering new treatments for genetic epilepsies. The integration of advanced gene-editing techniques and high-throughput screening platforms holds promise for accelerating the development of effective therapies for CDD and other neurological disorders. 

## 6. Trace Cannabinoids and Their Promising Anti-Epileptic Effects

Many of the minor trace cannabinoids show promise in their anti-epileptic effects and present interesting targets for future preclinical and clinical studies [125]. Cannabidivarin (CBDV), structurally similar to CBD, has shown efficacy as an anticonvulsant in PTZ and audiogenic-induced rat and mouse models [57,133,134]. THC acid showed mixed results in several induced seizure types DS mouse models and showed synergistic effects between THC and THCA [135], while Δ9-Tetrahydrocannabivarin was anticonvulsant in rats [133]. CBDV acid, CBG acid (CBGA), Cannabigerovarin (CBGV) acid, Cannabichromene (CBC), CBC acid, and Cannabichromevarin (CBCV) acid were all found to be in hyperthermia-induced seizures in the DS mouse model [136,137]. CBGA was able to potentiate the anti-convulsive effects of CLB, but monotherapy at high doses was proconvulsive. The cannabinoid biosynthesis precursor olivetolic acid was found to attenuate hyperthermia-induced seizures in the DS Scn1a+/− mouse model [138].

Mechanisms are still to be elucidated but appear to interact with receptors in similar pathways to CBD and THC. THCV and Cannabigerol (CBG) may act as an antagonist of CB1R [139,140] and 5-HT1A [140], opposing the agonism of CBD. But CBG acts with CBD, CBGV, and THCV as an agonist of TRPV1 [59]. CBGA is reported to have inhibitory effects on TRPM7 [141] and TRPM8 [142], cation channels that can inhibit seizure-like activity in vitro [143,144]. However, CBG did not reduce PTZ-induced seizures in rats [145]. CBGA interacts with the GPR55 receptor, TRPV1, and is a positive modulator of GABAR [137].

Other phytocannabinoids act as partial agonists of CB1R, including cannabinol (CBN) and Δ9-Tetrahydrocannabivarin (THCV). THCV may also act as an antagonist at low doses. Cannabigerol may act as an antagonist of CB1R [140].

Together, these trace cannabinoids may offer additional therapeutic benefits in the treatment of epilepsy, particularly in drug-resistant cases, and their interactions with each other and with major cannabinoids like CBD suggest a potential for synergistic effects that could enhance overall seizure control. Because some appear to have proconvulsant effects or may oppose the effects of other cannabinoids, it remains important to continue the research into these trace compounds and ensure their concentrations are known in medicinal products.

## 7. Conclusions

The landscape of cannabinoid research in the context of CDD presents both promises and challenges. While clinical trials and observational studies have shown promising results regarding the efficacy of CBD as an adjunct therapy for CDD-associated epilepsy, there is a clear need for more high-quality, controlled clinical trials specifically focused on CDD patients. The limited availability of such trials, coupled with challenges in standardizing cannabis-derived products, poses difficulties in assessing long-term efficacy.

Much attention has been paid to CBD, yet there has been little consideration for the potential synergistic effects of other cannabinoids or terpenes, which could either enhance CBD’s efficacy or pose risks. With the large number of potential cannabinoid combinations, conducting clinical trials in these small patient cohorts is impractical. Therefore, we call for the use of CDD epilepsy models in preclinical trials to uncover optimal companion AEDs. Researchers, clinicians, and caregivers must also prioritize the sourcing and accurate analysis of cannabis-derived products and carefully consider the off-label use of artisanal CBD compounds, which may lead to exclusion in future clinical trials. This approach can provide valuable data to guide future trials toward analyzing potentially beneficial compounds.

## 8. Future Directions 

First, it is crucial to examine the composition of cannabinoid extracts to fully understand the factors influencing their makeup and ensure consistency in potential medications. With the success of CBD clinical trials in other DEEs, there is a pressing need for high-quality, double-blind, placebo-controlled clinical trials specifically targeting CDD populations. These trials should include long-term follow-up to assess safety and effectiveness. 

Robust preclinical models should be employed to investigate the pharmacodynamics of CBD and other cannabinoids in CDD-specific epilepsy. Isolated cannabinoids and combinations should be evaluated for potential synergistic effects, particularly with trace cannabinoids or concomitant AEDs. Given the scarcity of CDD-specific preclinical trials, it is essential to develop CDD-specific disease models that can provide critical insights to guide future clinical trial designs.

Patient-derived cell models and zebrafish are promising systems for high-throughput drug screening in CDD epilepsy. In cell-based models, key endpoints for CDD may include multielectrode arrays to examine electrophysiology, calcium imaging, mitochondrial function, and oxidative stress assays. Additionally, the expression and localization of key epilepsy-related proteins, such as the NMDA, AMPA, and GABA receptors, and synaptic markers may be useful biomarkers. These are ideal systems for investigating the role of CDKL5 in epileptogenic and drug screening. It would also be of interest to examine the effect of CBD on CDD-specific endpoints, such as phosphorylation levels of known CDKL5 targets and cilia length. In zebrafish models, endpoints should include seizure frequency and behavioral phenotyping through hyperactivity or movement tracking. 

Modeling patient-specific genetic variants is valuable for evaluating the idiosyncratic effects of cannabinoids, enabling the development of personalized, effective treatment strategies.

## Abbreviations

Anti-Epileptic Drug (AED), CDKL5 Deficiency Disorder (CDD), Clobazam (CLB) Developmental and epileptic encephalopathy (DEE), Cannabidiol (CBD), Cannabinol (CBN), Cannabichromene (CBC), Cannabigerol (CBG), Cannabidivarin (CBDV), Δ9-Tetrahydrocannabivarin (THCV), Dravet syndrome (DS), Endocannabinoid system (ECS), Lennox–Gastaut Syndrome (LGS), protein phosphatase 1 (PP1), Δ9-tetrahydrocannabinol (THC), 2-arachidonoylglcerol (2-AG), N-arachidonoylethanolamide (AEA).
